# Technical approach and outcomes of failed infrarenal endovascular aneurysm repairs rescued with fenestrated and branched endografts

**DOI:** 10.1186/s42155-019-0075-z

**Published:** 2019-10-27

**Authors:** Jesse Manunga, Larissa I. Stanberry, Peter Alden, Jason Alexander, Nedaa Skeik, Elliot Stephenson, Jessica Titus, Joseph Karam, Xiaoyi Teng, Timothy Sullivan

**Affiliations:** 1 Department of Vascular and Endovascular surgery, Minneapolis Heart Institute Foundation, Abbott Northwestern Hospital, 920 E. 28th Street, Suite 300, Minneapolis, MN 55407 USA; 2 Minneapolis Heart Institute Foundation, Minneapolis, MN USA

**Keywords:** Aortic aneurysm, Failed EVAR, Rescue, Fenestrated/branched endografts, Inverted iliac limb

## Abstract

**Background:**

Endovascular rescue of failed infrarenal repair (EVAR) has emerged as an attractive option to stent graft explantation. The procedure, however, is underutilized due to limited devices accessibility and the challenges associated with their implantation in this patient population. The purpose of this study was to report our outcomes and discuss our approach to rescuing previously failed infrarenal endovascular aneurysm repairs (EVAR) with fenestrated/branched endografts (f/b-EVAR).

**Methods:**

A retrospective analysis of prospectively collected data of consecutive patients with failed EVAR rescued with f/b-EVAR at our institution from November 2013 to March 2019 was conducted. The study primary end point was technical success; defined as the implantation of the device with no type I a/b or type III endoleak or conversion to open repair. Secondary endpoints included major adverse events (MAEs), graft patency and reintervention rates.

**Results:**

During this time, 202 patients with complex aortic aneurysms were treated with f/b-EVAR. Of these, 19 patients (Male: 17, mean age 79 ± 7 years) underwent repair for failed EVAR. The median time from failed repair to f/b-EVAR was 48 (30, 60) months. Treatment failure was attributed to stent graft migration in 9 (47.4%) patients, disease progression in 5 (26.3%), short initial neck in 3 (15.8%) and unable to be determined in 2 (10.5%). Three patients were treated urgently with surgeon modified stent graft. Technical success was achieved in 18 patients (95%), including two who had undergone emergent repair for rupture. Seventy-two targeted vessels (97.3%) were successfully incorporated. Sixteen (84.2%) patients required a thoracoabdominal repair to achieve a durable seal. Major adverse events (MAEs) occurred in 3 patients (15.7%) including paralysis and death in one (5.3%), compartment syndrome and temporary dialysis in another and laparotomy with snorkeling of one renal and bypass of the other in the third patient. Median (IQR) hospital length of stay was 3 (2, 4) days. Late reintervention, primary target vessel patency and primary assisted patency rates were 5.3%, 98.6% and 100%, respectively.

**Conclusion:**

Implantation of f/b-EVAR in patients with failed previous EVAR is a challenging undertaking that can be performed safely with a high technical success and low reintervention rates.

## Background

One of the most dreaded long-term complications of EVAR remains the loss of proximal seal due to disease progression. Unfortunately, the vascular community is likely to see an increase number of these cases since our patients are living longer and the number of endovascular enthusiasts as well as the liberal use of EVAR, even in hostile necks, will only continue to rise with time. While some patients with this pathology can be treated with an infrarenal cuffs and endoanchors, others do not have a “neck” suitable for such interventions and require endografts explantation or extension of the repair above renal arteries. Stent graft explanation carries a mortality and morbidity rates as high as 24% and 65%, respectively (Perini et al., 2019; Arnaoutakis et al., 2019; Klonaris et al., 2014; Nabi et al., 2009; Pitoulias et al., 2009). Endovascular options for patients with failed EVAR include parallel grafts and fenestrated/branched endografts (f/b-EVAR). Parallel grafts have the advantage of using readily available, off-the-shelf components approved for other indications. However, the technique has a high incidence of gutter endoleaks (Scali et al., 2014; Lee et al., 2012; Donas et al., 2015).

F/b-EVAR can be customized to fit the patient’s anatomy and are increasingly being used outside of the United States (US) for this purpose (Katsargyris et al., 2013; Falkensammer et al., 2017). In the US however, the Food and Drug Administration (FDA) regulations limit the availability of these devices to centers with investigational device exemptions (IDE) (Martin et al., 2014). The purpose of this study was to report our experience and describe our approach to the endovascular management of failed EVAR using f/b-EVAR.

## Methods

The study is an Institutional Review Board (IRB) approved retrospective analysis of prospectively collected data of consecutive patients with failed EVAR treated with f/b-EVAR from November 2013 to March 2019 in the division of Vascular and Endovascular Surgery at Abbott Northwestern Hospital.

All but one included patients had at least two postoperative thin slice computer tomography scan (CTA). All CTAs were obtained in three phases and analyzed using TeraRecon (TeraRecon, Foster City, CA, USA) to obtain preoperative measurements for stent graft customization. Whenever available, initial scans and device implantation angiograms were analyzed to determine the cause of treatment failure and operative reports were reviewed. While no formal protocol was utilized, careful attention was paid to the aortic neck morphology, presence of any endoleaks on completion angiography, as well the device manufacturer, size and number of pieces used. Every attempt was made to determine the reason for primary treatment failure. The American Society of Anesthesiologists (ASA) physical classification and the Society for Vascular Surgery/American Association for Vascular Surgery (SVS/AAVS) comorbidity severity scores were calculated preoperatively and laboratory values were scrutinized pre and postoperatively.

Data collected included patient’s demographics, cardiovascular profile, radiologic data, additional stent graft modifications, procedure length, radiation dose, contrast medium volume, persistent endoleak, conversion to open repair, intensive care unit length of stay (ICU LOS), hospital length of stay (HLOS) and all procedure related major adverse events (MAEs). The study primary endpoint was technical success; defined as the successful implantation of f/b-EVAR with no type I a/b or type III endoleak or conversion to open repair. Secondary endpoints included MAEs, graft patency and reintervention rates. MAEs included procedure related stroke, any myocardial infarction, pneumonia, renal failure requiring dialysis, transient or permanent paraplegia, bowel ischemia, lower extremity compartment syndrome and death.

### Device customization

**Fig. 1:**
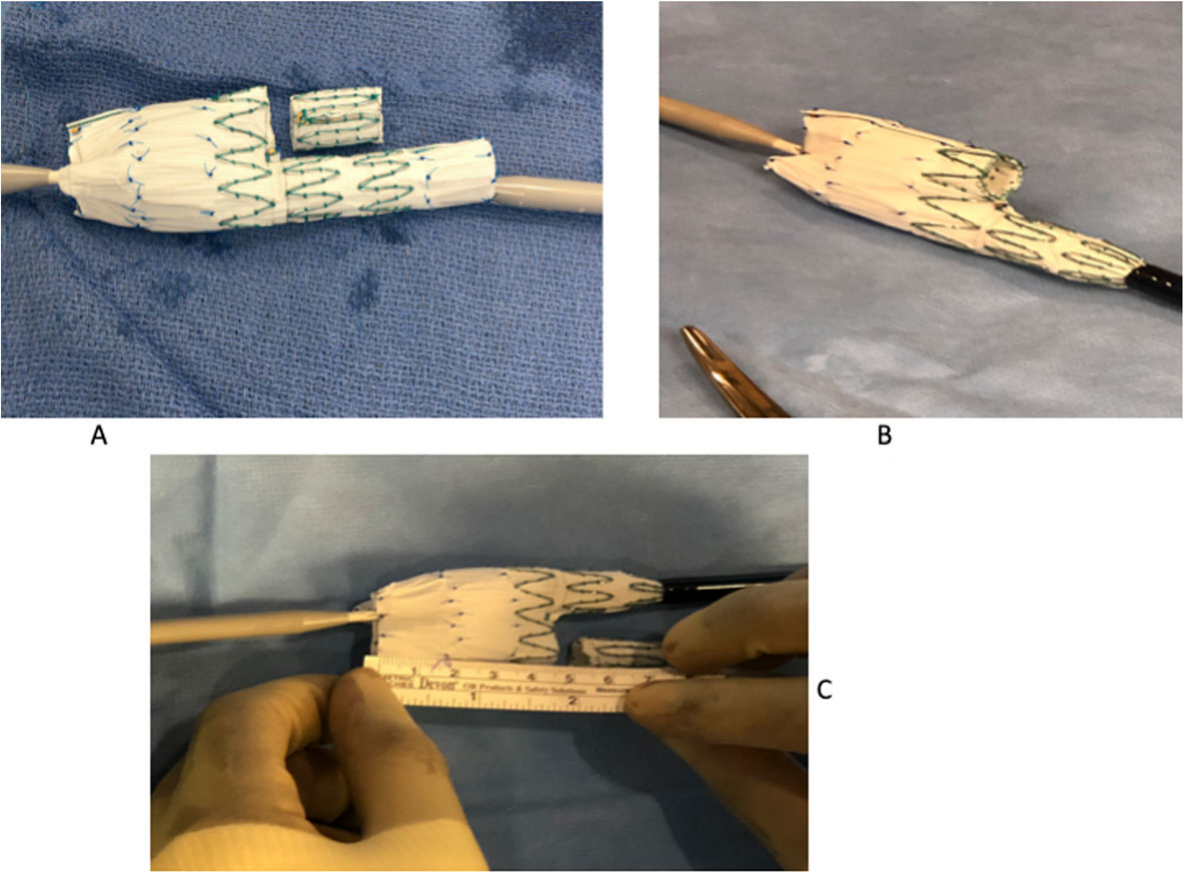
Creation of inverted iliac limb using the Cook Zenith Fenestrated distal bifurcated body stent graft. **a** Preparing the inverted limb. Note that the graft is partially deployed; the contralateral limb transected using an ophthalmologic cautery. The check mark is placed in the original orientation to facilitate gate cannulation. **b** Minimum distance to the flow divider post limb inversion. The length from the top of the stent graft to the gate has been reduced from 76 mm to 51 mm, allowing relining of the entire failed previous EVAR. **c** Securing the inverted limb to the bifurcated device. The transected limb is now inverted and sewn in place with a 5–0 double arm ethibond suture

**Fig. 2:**
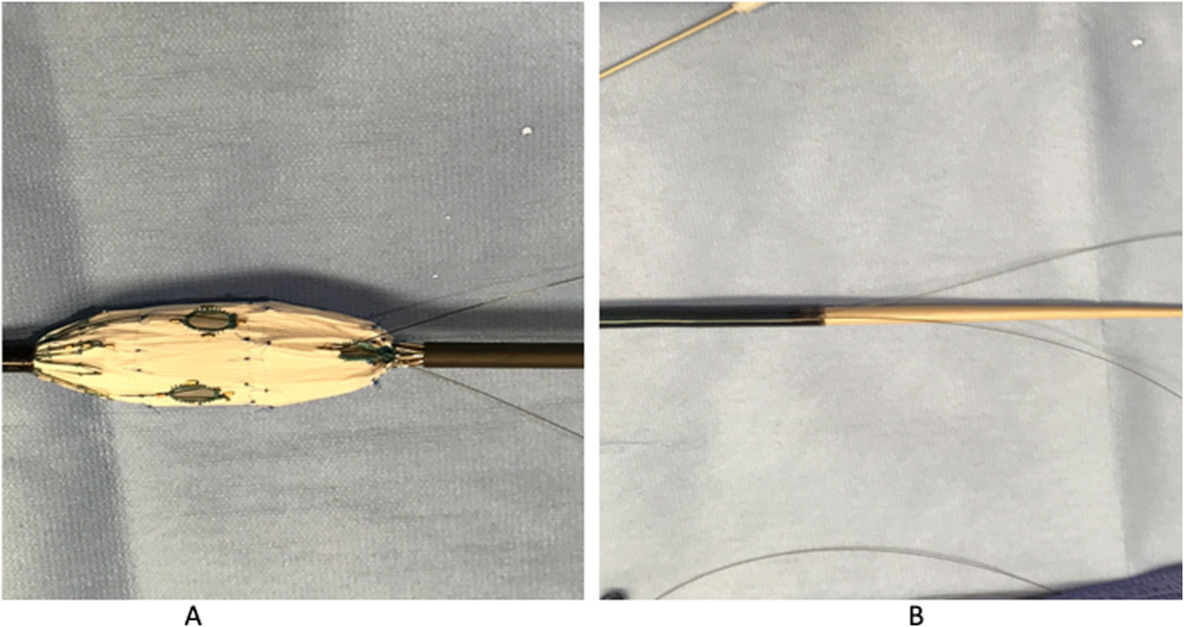
**a** Loading wires in a fenestrated device. The Cook Zen-fen proximal graft was ordered with 1 scallop to accommodate the SMA and 2 small fenestrations to accommodate renal arteries. The device is deployed on a sterile back table and a 0.014 and 0.018 long wires are placed through the scallop into the body of the fenestration device, out through the small (renal) fenestrations. **b** Re-sheathed the device after loading wires. The device is now re-sheathed and ready to be implanted. The preloaded wires allow for cannulation of target vessels and placement of bridging stents from the axillary access site prior to releasing constraining wires

All fenestrated stent grafts were customized using the Cook Zenith platform (Cook Medical, Bloomington, IN). Fenestrations were created based on measurements obtained using centerline of flow. Fenestrated “cuffs” were the preferred configuration for all patients as long as distal seal could be achieved inside the failed device with two or more sealing stents. In instances where seal could not be achieved, a combination of a fenestrated cuff and a bifurcated device with or without inverted contralateral limb was used to reline the entire infrarenal aorta and common iliac arteries. Inverted limbs were created by removing the stent of the bifurcated device’s contralateral limb, inverting it inside the gate and suturing the two pieces with a doubled arm 5–0 Ethibond suture in a locking fashion (Fig. 1 a, b & c). In a few instances, the distal stent of the Zenith Fenestrated proximal body (Z-fen) was removed with an ophthalmologic cautery to overcome the short working distance between the lowest renal artery and the flow divider of the failed EVAR. Additional fenestrations were added accordingly to allow for incorporation of all targeted vessels. Preloaded wires were used in the case of a patient with a failed aorto-uni-iliac device (AUI) to allow for cannulation of target vessels from the left axillary artery access site (Fig. 2 a & b). Three emergent cases were treated with surgeon-modified fenestrated stent grafts (SMFSG) using bifurcated zenith devices or Cook Alpha proximal thoracic stent graft. Two of the three patients had contained ruptured aneurysm. The third patient had a large aneurysm sac with type IA endoleak and complaining of dull back pain not believed to be aneurysm related. However, we believed he was too high risk for rupture to wait for a manufactured fenestrated device. SMFSG were modified as previously reported by Oderich (Ricotta 2nd & Oderich, 2008) and Manunga (Manunga, 2018).

### Device implantation and postoperative care

F/b-EVAR implantations were performed in a hybrid operating room under general anesthesia. Spinal drains were placed in all patients requiring 2 cm or more aortic coverage above the celiac artery. Our technical approach to implantation of these devices has been previously reported (Manunga, 2018; Manunga et al., 2018; Manunga & Titus, 2017). Since these reports, we have adopted the routine use of fusion technology, allowing us to abandon pre-cannulation of renal arteries or pre-implantation angiography. In the case of a failed AUI, the fenestrated device was designed with a scallop to accommodate the superior mesenteric artery and two small fenestrations for renal arteries. The fenestrated device was deployed on a sterile back table; two long wires (0.014 and 0.018) preloaded going through the scallop into the main body of the graft and out through small fenestrations (Fig. 2 b&c). Following a right femoral artery to a left axillary artery through-and- through access, the preloaded wires were fed into a catheter and advanced along with the fenestrated device loaded on a lunderquist wire. The device was deployed in the standard fashion and renal arteries catheterized using preloaded wires and bridging stent grafts placed.

Diet, for the majority of patients, was resumed on postoperative day number one. Most patients had a CTA prior to discharge to ascertain exclusion of the aneurysm and absence of any endoleaks, bridging stent kinking or branch malperfusion. Follow up consisted of a CTA, liver function test, and creatinine at 3 months, 6 months and yearly thereafter. In some cases, especially when the 3-month CTA does not show an endoleak, the 6-month studies are skipped.

### Statistical analysis

Patients demographic, clinical, and procedural characteristics were summarized using count (%) for categorical data, mean ± deviations for symmetrically distributed (skewed) continuous variables, and medians and interquartile ranges fro skewed data. The analysis was performed using R 3.5.2 in R-Studio 1.1.463 environment.

## Results

### Patient characteristics

Between November 2013 and March 2019, 202 patients with complex aortic aneurysms were treated with f/b-EVAR in our division. Of these, 19 (9.4%) underwent endovascular recue of failed EVAR. Two patients were treated emergently for rupture and 17 underwent elective repair. Seventeen patients (89.5%) were male with a mean age of 79 ± 7 years. Thirteen (68.4%) patients had family history of aneurysmal disease. Fifteen patients (78.9%) were classified ASA III and four as ASA IV. The mean SVS/AAVS comorbidity score was 16.2 ± 3.1 (Table 1). Seven patients with failed EVAR were deemed physiologically fit for open repair, underwent endograft explantation and were excluded from this analysis.

**Table 1:** Patient demographics and cardiovascular risk factors

Variables	All, *n* = 19
Age (years)	79 ± 7
Gender	n (%)
Male	17 (89)
Cardiovascular risk factors	n (%)
Coronary artery disease	19 (100)
Hypertension	19 (100)
Hyperlipidemia	19 (100)
Tobacco abuse (history of)	18 (95)
COPD	11 (58)
CHF	6 (32)
Cerebral vascular disease	14 (74)
Peripheral arterial disease	8 (42)
Diabetes Mellitus	3 (16)
Renal insufficiency (GFR < 30)	6 (31.6)
Family history of aneurysmal disease	13 (68.4)
ASA III	15 (78.9)
ASA IV	4 (21.1)
SVS/AAVS cormorbidity score	16.2 ± 3.1
Size (mm)	Mean, SD
Aortic neck^a^	32 ± 4
Aneurysm	74 ± 12
Time laps^b^ (months)	48 (30, 60)
Indication for intervention^c^	
Type IA endoleak	18 (94.7)
Type IA and B endoleak	1 (5.3)
Endotension	1 (5.3)

Legend: *COPD* chronic obstructive pulmonary disease, *GFR* Glomerular Filtration Rate, *SVS/AAVS* Society for Vascular Surgery/American Association for Vascular Surgery, *ASA* American Society of Anesthesiologists

^a^Aortic neck as measured just below renal arteries

^b^Time laps from initial EVAR to f/b-EVAR shown as medians (25th percentile, 75th percentile)

^c^All patients undergoing repair had an increase in aneurysm sac in the presence

### Aneurysms characteristics, previous repair and causes of primary repair failure

Sixteen patients (84.2%) were referred to us from outside institutions and deemed poor candidates for stent graft explantation. The mean aortic neck and aneurysm size prior to f/b-EVAR were 32 ± 4 mm and 74 ± 12 mm, respectively. The mean time from initial EVAR to f/b-EVAR was 48 (range: 30–60) months. Seventeen patients had an increase in the neck size (Tables 1 & 2). Eight (42.1%) were treated with an excluder (W.L. Gore & Associates, Flagstaff, AZ, USA), 9 (47.4%) with a Talent (Medtronic World medical, Sunrise, FL, USA) and 2 (10.5%) with a Cook Zenith stent graft (Cook Inc., Bloomington, IN, USA). Fourteen (73.7%) patients had undergone previous attempts at fixing endoleaks with 9 (47.4%) of these having had 2 or more interventions prior to f/b-EVAR (Table 2). Treatment failure was the result of stent graft migration in 9 patients, disease progression in 5, short initial neck (< 15 mm) in 3 and unable to determine in 2. Eighteen (94.7%) patients had type Ia endoleak. One patient did not have a clear endoleak, even on conventional angiogram, but had a > 33 mm increase in aneurysm sac size and was believed to have endotension (Table 3).

**Table 2:** Patient Characteristics and Preoperative Variables

Patient			Aneurysm size & previous intervention				
No	Age (yrs.)	Gender	Size (mm)	Neck (mm)	Device	TL (mo.)	Interventions
1	81	M	71	32	Gore	156	Aortic cuff, palmaz
2	76	M	77	28	Gore	108	Aortic cuff, coil embolization
3	83	F	84	23	Gore	36	Aptus × 2
4	63	M	78	36	Gore	60	Aptus × 1
5	72	M	62	36	Cook	109	IMA and lumbar embolization
6	72	M	65	32	Medtr	25	None
7	71	M	97	36	Medtr	37	Aptus, lumbar embolization
8	77	M	85	36	Gore	13	Aptus, cuff
9	71	M	72	23	Gore	61	Cuff, lumbar embolization
10	80	M	68	35	Gore	32	none
11	76	M	61	36	Medtr	47	None
12	92	M	78	30	Medtr	25	Aptus × 3, coil embolization
13	80	M	74	28	Medtr	85	Aptus × 2
14	81	F	83	26	Cook	23	None
15	77	M	80	36	Medtr	13	none
16	84	M	58	30	Medtr	49	Cuff, aptus
17	76	M	56	32	Gore	53	None
18	89	M	88	36	Medtr	49	Aptus, Palmaz
19	86	M	94	32	Medtr	37	Cuff, aptus, IMA embolization

Legend: *Medtr* Medtronic, *IMA* inferior mesenteric arery, *TL* time from initial EVAR procedure to f/b-EVAR implantation, *cuff* aortic cuff

**Table 3:** Presumed causes for primary EVAR failure

Causes of primary treatment failure	Number of patients (%)
Stent graft migration	9 (47.4)
Disease progression	5 (26.3)
Short initial neck	3 (15.8)
Unable to determine	2 (10.5)
Total	19 (100)

### Fenestrated/branched device configurations

Twelve (63.2%) patients required a proximal fenestrated cuff and a distal bifurcated device to successfully exclude the aneurysm since the existing device was bigger than the fenestrated piece implanted. Of these, five needed creation of an inverted limb in order to overcome the limited working length within the existing graft. Five patients (26.3%) were treated with a fenestrated cuff alone. One patient required a 4-vessels fenestrated stent graft and a Gore excluder iliac branch endoprosthesis (IBE) to address a concomitant type Ib endoleak. Device configuration consisted of 4 fenestrations in 16 patients (57 small fenestrations, 15 large fenestrations and 1 scallop). Three patients required 3 vessels fenestrated devices. Overall, 73 fenestrations or scallop and 1 branch were needed to incorporate 74 target vessels in all 19 patients (Table 4 & 5).

**Table 4:** Fenestration type, vessels targeted and vessels successfully incorporated

Incorporated vessel	Small fenestration	Large fenestration	Scallop	Branch	Vessels successfully incorporated (%)	Total(%)
Renal artery	38	0	0	0	36 (95)	38 (51.4)
SMA	16	3	0	0	19 (100)	19 (26.7)
Celiac artery	3	12	1	0	16 (100)	16 (21.6)
Iliac artery	0	0	0	1	1 (100)	1 (1.3)
Total	57	15	1	1	72 (97.3)	74 (100)

*SMA* superior mesenteric artery

**Table 5:** Operative and postoperative variables

Variables	All, *n* = 19, (%)
Devices	
Proximal ZFen + bifurcated + iliac limbs	12 (63.2)
* Bifurcated with Inverted iliac limbs*	*5*
* Bifurcated without inverted limbs*	*7*
Fenestrated Cuffs alone	5 (26.3)
* Proximal Z fen cuff*	*4*
* Surgeon-modified Alpha*	*1*
Surgeon-modified bifurcated Zenith Fenestrations	2 (10.5)
4 fenestrations –TAR/supraceliac repair	16 (84.2)
3 fenestrations/scallop – infraceliac repair	3 (15.8)
Fluoroscopy	
Time (minutes, SD)	70 ±25
Dose (mGy, SD)	3200 ±950
Median EBL (25th, 75th percentile)	100 (100, 200)
Procedure length (minutes, SD)	103 ±28
MAEs	
Renal Failure	2 (10.5)
Temporary dialysis	1 (5.3)
Permanent dialysis	1 (5.3)
Paraplegia	1 (5.3)
Compartment syndrome	1 (5.3)
Death	1 (5.3)
ICU LOS (days)	0 (0,0.5)
H LOS (days)	3 (2, 4)
Endoleaks	n (%)
Type Ia/b	0 (0.0)
Type II	3 (15.8)
Type III	0 (0.0)
Median follow-up (months) (25th, 75th percentile)	13 (5, 23)
Reintervention	n (%)
Early	2 (10.5)
Mid-term	1 (5.3)
Total	4 (15.8)
Patency^a^	n (%)
Primary	68 (98.6)
Primary assisted	69 (100)

*EBL* estimated blood loss; ^a^Patency of target vessels in 18 patients who survived initial hospitalization; *TAR* thoracoabdominal repair, *ICU LOS* intensive care unit length of stay, *HLOS* hospital length of stay

### Operative details

Operative repairs were performed via bilateral femoral artery access in 18 patients and a femoral and left axillary artery access in one patient. Devices were implanted successfully in 18 patients (95%). In one patient, renal arteries were cannulated with glide wires but sheaths needed for the delivery of the bridging stent could not be advanced. Various maneuvers were attempted to no avail. The right renal artery was snorkeled and the left surgically bypassed. Overall, 74 vessels were targeted and 72 (97.3%) were successfully stented, 2 fenestrations could not be stented and the scallop was intentionally not stented.

All successfully cannulated target vessels were bridged using iCAST atrium covered stents (Maquet, Germany) with the exception of the right internal iliac artery where a viabahn VBX (L.W. Gore, Flagstaff, AZ, USA) was used. In two patients, one renal artery could not be stented during the index procedure. However, kidneys were perfused on angiography and decision was made to complete repair at another time. Both patients were successfully stented during subsequent operations using a brachial approach.

### Intraoperative technical issues and 30-day outcomes

Difficulty in lining up fenestrations was encountered in 9 (47%) patients and resulted in removal of the devices from the body, rotation outside of the body anywhere from 90 to 180 degrees from what would have been the normal delivery position. In some patients, cannulation of renal arteries was particularly challenging. This led to two patients requiring a secondary procedure and one laparotomy.

Three patients suffered MAEs, including development of bilateral lower extremity compartment syndrome requiring fasciotomy and subsequent short-term dialysis in one, post-operative paraplegia and death in another and a laparotomy with snorkeling of the right renal artery and surgical bypass of the left renal artery in the third. The third patient returned to the operating room to revise the thrombosed renal artery bypass and has been on dialysis for a little over 2 months although she has started producing urine (Table 5). The median hospital length of stay was 3 (2, 4) days. Thirteen patients (72.2%) were discharged home; four (21.1%) required short-term rehabilitation stay and one died 4 days post f-EVAR.

### Follow-up and long-term survival

The mean follow-up was 13 (range: 5–23) months. Aneurysm sac regression occurred in 16 patients (84.1%). Target vessels have remained patent in all who survived the index hospitalization. However, one patient developed chronic mesenteric ischemia due to a high-grade stenosis of the SMA bridging stent. It was successfully ballooned with resolution of his symptoms. The primary patency rate of successfully stented vessels in the 18 patients who survived the repair was 98.6% and primary assisted patency was 100%. Type II endoleaks resolved in 2 patients within the first year post f/b-EVAR but persists in one patient. One patient died of metastatic lung cancer 39 months post f-EVAR bringing the overall survival to 89.5% (Table 5).

## Discussion

F/b-EVAR arguably represents the best endovascular option for patients with failed previous EVAR. However, this technique can be challenging for several reasons. First, the working length between the lowest renal artery and the flow divider of the existing graft is often too short to accommodate currently approved Z-fen configurations. Second, discrepancy in size between the failed device and the fenestrated device often makes achieving a seal problematic. Third, the ability to rotate and accurately deploy the fenestrated device inside the patient can be severely compromised by vessel tortuosity and friction between devices. Lastly, cannulation of target vessels can be difficult, especially in patients with suprarenal fixation. These challenges, in part, accounted for the higher than our previously reported average fluoroscopy time (61 min) and dose (1097 mGy) with this procedure (Manunga et al., 2018).

Twelve (62.3%) patients in our cohort required complete relining (Z-fen+ bifurcated device + iliac limbs) in order to achieve a seal. This process was facilitated by creation of an inverted limb in 5 patients. While the technique of inverted limb has previously been described (Martin et al., 2014; Jain et al., 2016), two key differences deserve mentioning. First, devices used in recent series were custom-made by the industry while inverted limbs used in our cohort were surgeon-modified. Second, the main body of industry-made devices has two sealing stents whereas our devices had three sealing stents and, therefore, allowed for a longer overlap. Creation of an inverted limb shortens the distance between the top of the graft and the gate of the shortest distal cook bifurcated main body graft from 76 mm to 51 mm. Using this technique in a patient with a distance from the lowest renal artery to the flow divider of < 49 mm may result in “jailing” of the gate.

While not utilized in this cohort, alternatives to inverted limb include the use of an AUI device or a Gore IBE. The use of AUI devices has a disadvantage of long-term femoral to femoral bypass graft occlusion and decreased pelvic flow. The IBE has a main body diameter of 23 mm and a distance from the top of the graft to the gate of 55 mm. If one opts on using this device, the repair needs to be built from the bottom up – the IBE is placed first, followed by implantation of the fenestrated device – to obtain a seal since the distal fenestrated device is 24 mm in diameter.

Rescuing a failed AUI device with f-EVAR is feasible and requires the use of preload wires on the fenestrated device. We prefer using the 65 cm, 12 French Gore DrySeal (L.W. Gore, Flagstaff, AZ, USA) in the axillary artery and cannulating target vessels from the arm access. We do not advocate releasing diameter reducing ties before securing target vessels as doing so would almost certainly make cannulation of target vessels extremely difficult, if not impossible.

Converting various devices to a fenestrated repair present different set of challenges. The distance between the top of the graft to the flow divider in a Gore excluder is device size dependent and ranges from 40 to 60 mm. In our experience, a failed excluder tends to migrate down, allowing for ≥ 20 mm in additional working length. This makes complete device relining feasible in most cases. Medtronic Talent and Cook endografts have longer main bodies and tend to migrate less. However, they have suprarenal struts that might make placement of bridging stents difficult. This was the case with the single patient in our cohort that required a laparotomy.

Our treatment philosophy in younger patients, those with failed EVAR or family history of aneurysmal disease differs from that of patients without this history. In the above population, we strive to maximize the aortic neck by incorporating all 4 visceral arteries whenever feasible. However, the need for a longer sealing zone needs to be balanced with the risks of paraplegia. We routinely analyze and minimize the number of intercostal arteries covered by the repair in order to mitigate the risk of paraplegia. Furthermore, we strive to keep lower extremity ischemia time short and use spinal drains and neuromonitoring in all patients undergoing > 2 cm coverage of the aorta above the celiac artery.

Understanding the reason for primary treatment failure is crucial. In the series from Katsargyris et al., EVAR failure in all 26 patients was attributed to low initial stent-graft implantation in 27% of patients, short initial neck in 19%, undersized initial stent-graft in 8%, stent graft migration and disease progression in 23%, respectively (Katsargyris et al., 2013). In the Austria series, treatment failure was attributed to type I endoleak in 58.3% patients, stent graft migration in 16.7% and disease progression in 25% (Falkensammer et al., 2017). In the Cleveland clinic series, treatment failure was attributed to type IA endoleak in 70.4% of patients, stent migration in 33.3% and neck degeneration in 14.8% with some patients having a combination of these factors (Falkensammer et al., 2017). Wang et al. attributed treatment failure in a series of 12 patients to neck enlargement after open repair in 6, type IA endoleak in 5 and neck enlargement in 1 patient post EVAR (Wang et al., 2018). In our series, type IA endoleak was observed in 94.7% of patients with stent graft migration accounting for 47.4% of primary treatment failure, disease progression for 26.3% and short neck for 15.8%. The underlying theme of the above studies remains the same – most EVARs fail because of loss of proximal seal, which is the consequence of disease progression or implantation of the device in a hostile neck.

Sixteen patients (84.2%) in this cohort required a thoracoabdominal repair with spinal drain and neuromonitoring. Even with this complex reconstruction, most patients were discharged home within 3 days of their surgery. This, in our opinion, validates the advantage of endovascular intervention over open surgical repair in this patient population (Perini et al., 2019; Arnaoutakis et al., 2019; Klonaris et al., 2014; Nabi et al., 2009)^.^

MAEs occurred in three patients (15.7%). The first patient underwent a successful exclusion of the aneurysm but developed bilateral lower extremity compartment syndrome overnight and required fasciotomies. He was on dialysis for 2 weeks prior to normalization of his renal function. The second patient was an octogenarian with a ruptured aneurysm treated with a 3 vessel fenestrated device. He developed paraplegia postoperatively after suffering an episode of hypotension. A spinal drain was placed, all antihypertensive medications discontinued and mean arterial pressure (MAP) raised with no improvement. He required reintubation because of fluid overload and expired shortly thereafter. The last patient required a laparotomy due to inability to advance sheaths needed to place bridging stents. This was likely due to the presence of suprarenal struts spanning the orifice of renal arteries that prevented passage of anything bigger than a 0.035 wire.

The inability to cannulate renal arteries was a common issue with this procedure and is likely related to access vessels tortuosity, suprarenal struts and the friction between the fenestrated device and the failed implant. In the Cleveland Clinic experience, technical success rate was 85%; early mortality 3.8% and target vessel perfusion rate was 92%. Seven patients lost their kidneys due to inability to cannulate renal arteries. Another patient lost a celiac artery due to dissection [10]. There were no early deaths in the Katsargyris et al. cohort. However, difficulty in target vessel catherization was encountered in 23.1% of patients, resulting in the loss of 4 target vessels and a successful cannulation rate was 94.6% (Katsargyris et al., 2013). In the series from Austria, technical success rate was 58.3%. Two celiac arteries were lost during cannulation but both remained asymptomatic (Falkensammer et al., 2017). Our technical success rate of 95%, target vessels incorporation of 97.3%, early (in hospital) and late reintervention rate of 15.5% and 5.3%, respectively, long-term target primary vessel patency of 98.6% and primary assisted patency rate of 100% compares favorably to results from these series (Katsargyris et al., 2013; Falkensammer et al., 2017; Martin et al., 2014; Wang et al., 2018). The single death in our series occurred in a patient treated for a rupture. The above three reports included only electively treated patients and, to our knowledge, none of the currently published series on this topic included patients treated on an emergency basis.

The use of surgeon-modified devices remains an important part of any aortic center as industry-made custom devices take weeks to manufacture and off-the-shelf devices currently being investigated only fit limited patients’ anatomy. Modification of the alpha graft can be challenging due to the presence of laser-cut proximal barb that prohibits retrograde resheathing of the device. Some people have resorted to cutting proximal barbs during modification. However, doing so might compromise the integrity of the device. Instead, we described a technique of transitioning the modified alpha graft through a series of peel away sheaths prior to loading it into its original sheath (Manunga, 2018). This approach remains necessary when treating aortic arch pathologies. When used to treat thoracoabdominal aneurysms or failed EVAR, we found it easier to use the newly released 65 cm Gore DrySeal sheath to deliver the surgeon-modified alpha device. In this case, the device is only transitions through one peel away sheath before going into the previously place Gore DrySeal sheath. Once in place, the sheath is pulled back to deploy the device. This technique eliminates the challenging step of introducing the modified Alpha stent graft into its original sheath.

The current study has several limitations. First, f/b-EVAR is only one of two endovascular options for failed EVAR. The use of parallel grafts, especially when incorporating 1 or 2 vessels, has an important role in the treatment of these patients (Donas et al., 2015). This is particularly true in the United States and other parts of the world where access to device customization is limited. Second, our center has a good experience with f-EVAR as a large number of patients have been treated with this technology over the last 5 years. As such, our results might not be reproducible. Nonetheless, our experience is unique in several ways. First, 84% of included patients underwent a thoracoabdominal repair. Second, the study included both elective and emergently treated patients. Third, we did not rely on the industry for further device customization. Instead, we ordered device within the current FDA regulations and slightly modified them to fit the purpose.

## Conclusion

Results of our series suggest that implantation of f/b-EVAR in patients with failed EVAR is safe, effective and can be performed with a good technical success rate. In our experience, the procedure is technically more challenging than implantation of fenestrated devices in native aorta and should likely be performed at designated high-volume centers with an experienced team.

## Data Availability

The datasets used and/or analyzed during the current study are available from the corresponding author on reasonable request.

## References

[CR1] Arnaoutakis DJ, Sharma G, Blackwood S, Shah SK, Menard M, Ozaki CK (2019). Strategies and outcomes for aortic endografts explantation. J Vasc Surg.

[CR2] Donas KP, Telve D, Torsello G, Pitoulias G, Schwindt A, Austermann M (2015). Use of parallel grafts to save failed prior endovascular aortic aneurysm repair and type Ia endoleaks. J Vasc Surg.

[CR3] Falkensammer J, Taher F, Uhlmann M, Hirsch K, Strassegger J, Assadian A (2017). Rescue of failed endovascular aortic aneurysm repair using the fenestrated Anaconda device. J Vasc Surg.

[CR4] Jain V, Banga P, Vallabhaneni R, Eagleton M, Oderich G, Farber MA (2016). Endovascular treatment of aneurysms using fenestrated-branched endografts with distal inverted iliac limbs. J Vasc Surg.

[CR5] Katsargyris A., Yazar O., Oikonomou K., Bekkema F., Tielliu I., Verhoeven E.L.G. (2013). Fenestrated Stent-Grafts for Salvage of Prior Endovascular Abdominal Aortic Aneurysm Repair. European Journal of Vascular and Endovascular Surgery.

[CR6] Klonaris C, Lioudaki S, Katsargyris A, Psathas E, Kouvelos G, Doulaptsis M (2014). Late open conversion after failed endovascular aortic aneurysm repair. J Vasc Surg.

[CR7] Lee JT, Greenberg JI, Dalman RL (2012). Early experience with the snorkel technique for juxtarenal aneurysms. J Vasc Surg.

[CR8] Manunga J (2018). Endovascular repair of aortic arch aneurysm with surgeon-modified fenestrated stent graft. Aorta (Stamford).

[CR9] Manunga J, Sullivan T, Garberich R, Alden P, Alexander J, Skeik N (2018). Single-center experience with complex abdominal aortic aneurysms treated by open or endovascular repair using fenestrated/branched endografts. J Vasc Surg.

[CR10] Manunga J, Titus J (2017). Regional anesthesia as the anesthetic of choice for high-risk surgical patients undergoing repair of juxtarenal aortic aneurysms with fenestrated stent grafts. J Vasc Surg.

[CR11] Martin Z, Greenberg RK, Mastracci TM, Eagleton MJ, O’Callaghan A, Bena J (2014). Late rescue of proximal endografts failure using fenestrated and branched devices. J Vasc Surg.

[CR12] Nabi D, Murhy DH, Park J, Zarins CK (2009). Open surgical repair after failed endovascular aneurysm repair: is endografts removal necessary?. J Vasc Surg.

[CR13] Oderich GS (2010). Technique of adding a diameter-reducing wire to the modified TX2 fenestrated stent graft. Vascular..

[CR14] Perini P, Gargiulo M, Silingardi R, Piccinini E, Capelli P, Fontana A (2019). Late open conversions after endovascular abdominal aneurysm repair in an urgent setting. J Vasc Surg.

[CR15] Pitoulias GA, Schulte S, Donas KP, Horsch S (2009). Secondary endovascular and conversion procedures for failed endovascular abdominal aortic aneurysm repair: can we still be optimistic?. Vascular.

[CR16] Ricotta JJ, Oderich GS (2008). Fenestrated and branched stent grafts. Perspect Vasc Surg Endovasc Ther.

[CR17] Scali ST, Feezor RJ, Chang CK, Waterman AL, Berceli SA, Huber TS (2014). Critical analysis of results after chimney endovascular aortic aneurysm repair raises cause for concern. J Vasc Surg.

[CR18] Wang SK, Drucker NA, Sawchuk AP, Lemmon GW, Salsing MC, Motaganahalli RL (2018). Use of the zenith fenestrated plateform to rescue failing endovascular and open aortic reconstructions is safe and technically feasible. J Vasc Surg.

